# Ab Initio Study of Graphene/hBN Van der Waals Heterostructures: Effect of Electric Field, Twist Angles and p-n Doping on the Electronic Properties

**DOI:** 10.3390/nano12122118

**Published:** 2022-06-20

**Authors:** Simone Brozzesi, Claudio Attaccalite, Francesco Buonocore, Giacomo Giorgi, Maurizia Palummo, Olivia Pulci

**Affiliations:** 1 Dipartimento di Fisica and INFN, Universitá di Roma Tor Vergata, Via della Ricerca Scientifica 1, 00133 Rome, Italy; maurizia.palummo@roma2.infn.it; 2 Centre Interdisciplinaire de Nanoscience de Marseille UMR 7325 Campus de Luminy, CNRS/Aix-Marseille Université, CEDEX 9, 13288 Marseille, France; attaccalite@cinam.univ-mrs.fr; 3 Energy Technologies and Renewable Sources (TERIN) Department, Italian National Agency for New Technologies, Energy and Sustainable Economic Development (ENEA), Casaccia Research Centre, 00123 Rome, Italy; francesco.buonocore@enea.it; 4 Department of Civil & Environmental Engineering (DICA), Universitá degli Studi di Perugia, Via G. Duranti 93, 06125 Perugia, Italy; giacomo.giorgi@unipg.it; 5 CNR-SCITEC, 06123 Perugia, Italy

**Keywords:** graphene, h-BN, Van der Waals heterostructures, density functional theory, THz nanodevices

## Abstract

In this work, we study the structural and electronic properties of boron nitride bilayers sandwiched between graphene sheets. Different stacking, twist angles, doping, as well as an applied external gate voltage, are reported to induce important changes in the electronic band structure near the Fermi level. Small electronic lateral gaps of the order of few meV can appear near the Dirac points K. We further discuss how the bandstructures change applying a perpendicular external electric field, showing how its application lifts the degeneracy of the Dirac cones and, in the twisted case, moves their crossing points away from the Fermi energy. Then, we consider the possibility of co-doping, in an asymmetric way, the two external graphene layers. This is a situation that could be realized in heterostructures deposited on a substrate. We show that the co-doping acts as an effective external electric field, breaking the Dirac cones degeneracy. Finally, our work demonstrates how, by playing with field strength and p-n co-doping, it is possible to tune the small lateral gaps, pointing towards a possible application of C/BN sandwich structures as nano-optical terahertz devices.

## 1. Introduction

The exfoliation of graphene, a single layer of graphite sheet in 2004 and the successive Nobel Prize in Physics to Geim and Novoselov (2010), mark the birth of the 2D materials (2DM) age [1,2,3,4]. Since then, a multitude of one or few layers atomic thick materials, with different mechanical, electronic, magnetic, and optical properties is under focus, being interesting both at a fundamental and applicative point of view [5,6,7,8,9,10,11,12,13] Beyond the study of 2DM monolayers [14], a very exciting development in the field is the possibility to stack them, like the bricks of an atomic-scale Lego play, in artificial Van der Waals heterostructures (vdW HTs), with strong covalent bonds within the atomic layers and weak vdW interaction among them, whose resulting properties can be, in principle, controlled, tuned, and manipulated on demand [15,16]. In this context, vdW HTs composed of graphene and other 2DM are largely investigated. Indeed, despite graphene having unique chemical and physical properties, where surely the most peculiar one is the presence of Dirac-like linear band dispersion, its semi-metallic nature prevents the use in several nanoelectronics and photonics’ devices. The realization of HTs [17] is then one of the explored strategies to open the gap of graphene but to keep, as much as possible, high electronic carrier mobility values [18,19,20]. Due to similar atomic structure and almost perfect lattice match [21,22], hexagonal boron nitride (h-BN) has been used in combination with graphene to build up new HTs, from bilayer to multilayer forms [23,24,25,26,27,28,29]. With h-BN being an insulator with a wide band-gap of about 6 eV [30,31,32] and due to the large difference in the work functions of the two materials, a type-I band-alignment occurs. The resulting electronic properties are roughly the sum of those of the two separated systems, with low-energy graphene states falling always in the gap of h-BN. Nevertheless, as we show here and consistently with existing literature, the specific features near the Fermi level are strongly influenced by the specific stacking among graphene and h-BN sheets.

Motivated by interesting physical properties such as being a good platform for exciton condensation [33], tunable metal-insulator transition [34], observation of highly confined and topological plasmons modes [27,35] and possible applications in opto-electronic terahertz devices [36,37,38], we focus here on graphene/h-BN HTs, where two boron-nitride layers are inserted between two graphene ones. Our aim is to discuss how stacking order, twisting angle, external vertical gate voltage, and doping of graphene layers modify the electronic properties with respect to those of an isolated graphene sheet. The results presented here are obtained by means of first-principles Density Functional Theory (DFT) calculations. The analysis of the optical properties, as well as of the role of many-body effects, such as electronic self-energy and e-h interaction, will be the subject of future work.

## 2. Materials and Methods

All DFT calculations reported in this manuscript are obtained using the Quantum ESPRESSO suite [39,40,41] with plane wave expansion and scalar-relativistic norm-conserving pseudopotentials [42,43,44]. A kinetic energy cutoff of 60 Ry is adopted. PBE exchange–correlation [45] with vdW-DF2-b86r [46,47] exchange functional is exploited in order to take into account Van der Waals interaction. For the sake of comparison, we also perform several calculations of the structural properties using LDA exchange–correlation functional. A uniform Monkhorst–Pack k-point mesh of 60×60×1 is used for the non twisted systems, whereas a mesh of 12×12×1 is used for twisted HT. We introduce a vacuum of 30 Å in the non-periodic direction (z) to ensure periodic replicas’ decoupling. In order to simulate *n* and *p* doping of the graphene layers, a set of modified pseudopotentials are used, where C (Z = 4) atoms are substituted by C${}_{p}$ pseudo-atoms with Z = 3.98 (p doping) or C${}_{n}$ atoms with Z = 4.02 (n doping). In this way, we introduce an infinitesimal electronic negative charge in the top graphene layer (+0.02 electrons for each C${}_{n}$ atom) and an excess positive charge in the bottom one (−0.02 electrons for each C${}_{p}$ atom), still preserving the total charge neutrality of the system. In the non-twisted case, all the C atoms in the unit cell have been modified. In the twisted case, only four of the twenty-eight C atoms in the unit cell have been substituted (two with C${}_{p}$ atoms in the bottom graphene layer, and two with C${}_{n}$ in the top graphene layer). This choice determines different doping levels in the two cases.

Structure relaxation is assumed at convergence when the maximum component of the residual forces on the ions is smaller than $10^{-4}$ Ry/Bohr. Thanks to the small lattice mismatch of ∼1.5% between the two separated systems, we devise all the considered HTs using as a starting lattice parameter the average of the two lattice parameters and then fully relaxing the cell to find the equilibrium in-plane *a* lattice constant and inter-planar distances. Results of the structure optimization calculations confirm that the average of the lattice parameter was a valid guess.

## 3. Results

### 3.1. Structural Properties and Stacking Energies

**Figure 1:**
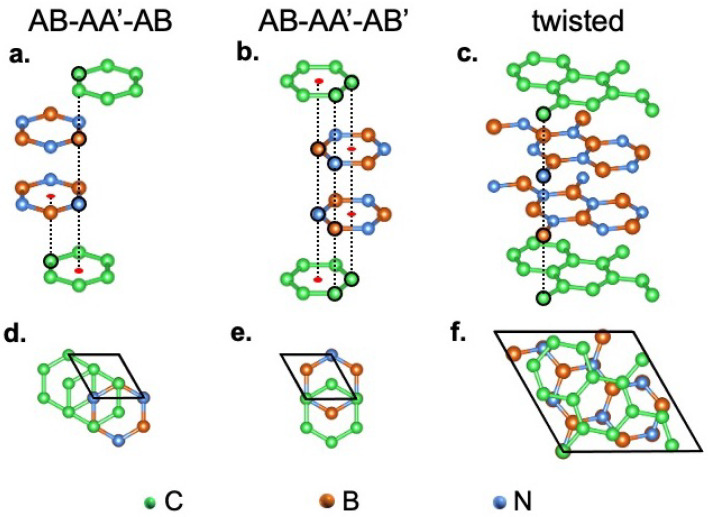
Crystal structures of C-BN-BN-C quadrilayers: (**a**,**d**) top and side view of AB-AA${}^{\prime }$-AB stacking. (**b**,**e**) the same for AB-AA${}^{\prime }$-AB${}^{\prime }$ stacking; (**c**,**f**) top and side view of the 21.8${}^{\circ }$ twisted case T-AA${}^{\prime }$-T. In the side views, the stacked atoms are circled in black, whereas the red dots indicate the vertical alignment of one atom in the upper layer with the center of the hexagon in the bottom layer.

In this section, we present the structural and energetic properties of three designed C-BN-BN-C quadrilayers, whereas the electronic properties are discussed in the next one. The atomic structures are shown in Figure 1, where the selected nomenclatures, AB-AA${}^{\prime }$-AB, AB-AA${}^{\prime }$-AB${}^{\prime }$, and T-AA${}^{\prime }$-T (T stands for twisted), are based on the stacking order of the central BN-BN and external C-BN interfaces. In the AA and AA${}^{\prime }$ configuration, a hexagon in one plane is on top of a hexagon in the plane below, whereas, in the AB Bernal type configuration, the hexagons are shifted, with one atom in the upper plane aligned to the center of the hexagon in the lower plane. Since 2D-hBN has two different atoms in the unit cell, two AA patterns are feasible: simple AA stacking, with B on B and N on N, and AA${}^{\prime }$, with B on N and vice versa. Preliminary stability analysis of the bilayers was performed in order to determine the lower energy configuration for HT interfaces. In particular, we calculate the stacking energies $\Delta E^{st}$ of bilayers and of quadrilayers, with $\Delta E^{st}=E_{tot}-\sum_{i}E_{i}$, where E${}_{tot}$ and $E_{i}$ are the HT and *i*-th mono-layer total energy, respectively [48,49,50,51,52].

Our study reveals that AB stacking for C/BN interface and AA${}^{\prime }$ stacking for BN/BN interface are the most stable configurations. As shown in Table 1, C-BN interface in AB stacking, with C on top of B, is more stable than AB${}^{\prime }$, with C on top of N, by ∼24 meV/atom. The same analysis for BN interface shows that the AA${}^{\prime }$ is the more stable stacking. For what concerns the quadrilayers, in the first HT (AB-AA${}^{\prime }$-AB, left panels of Figure 1), two completely equivalent C/BN (AB) interfaces are present, half of the carbon atoms of the top (bottom) graphene layer located above (below) boron atoms, and half at the center of h-BN hexagons. In the second one (AB-AA${}^{\prime }$-AB${}^{\prime }$, central panels of Figure 1), two non-equivalent C/BN interfaces occur, one AB${}^{\prime }$ (top) and the other AB (bottom): while, for the bottom graphene layer, the situation is similar to the previous case, for the top layer, half carbon atoms are located above the nitrogen atoms and half at the centers of hexagonal h-BN rings.

The twisted HT (T-AA${}^{\prime }$-T) is devised rotating clockwise by 21.8${}^{\circ }$ the two external graphene layers with respect to the two internal BN layers and has a $\sqrt{7}\times \sqrt{7}$ unit cell. Each layer contains 14 atoms. In the twisted HT, as evident looking at the right panels of Figure 1, only a limited number of C atoms are below or above B or N, and mixed types of stacking are present. In Table 1, we compare the stacking energies $\Delta E^{st}$ of the three quadrilayers: all considered structures show a gain in energy with respect to their monolayer counterparts, with the AB-AA${}^{\prime }$-AB HT the most stable, and the twisted the least stable one. $\Delta E^{st}$ of bilayers are also reported for comparison.

The equilibrium structural data of the three quadrilayers, together with those of graphene, BN monolayer, BN-BN, and C-BN bilayers, are listed in Table 2. Structural optimizations confirm the initial guess for lattice parameters of C-BN and C-BN-BN-C as being the average of those of single monolayers. The interplanar distances are slightly affected by the numbers of layers in the HT, especially for internal BN-BN AA${}^{\prime }$ layers. In fact, while graphene/boron-nitride distance is the same in C-BN and C-BN-BN-C, the distance between the two BN layers decreases from 3.30 Å (BN-BN freestanding) to 3.23(3.26) in twisted (AB-AA${}^{\prime }$-AB${}^{\prime }$) due to the interaction between the p${}_{z}$ orbitals of B/N atoms and those of carbon atoms in the adjacent graphene layer. Our results are in agreement with those reported by Giovannetti et al. [53], where a C-BN-BN 3-layer structure has been considered. Test calculations using LDA exchange–correlation provided in-plane lattice parameters very similar to PBE+vdW but slightly shorter inter-planar distances.

### 3.2. Electronic Properties

**Figure 2:**
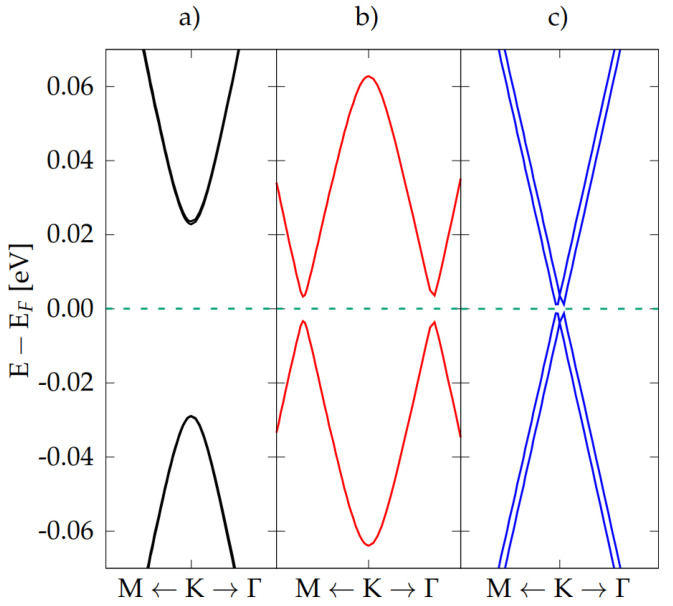
Band structure of the quadrilayers C-BN-BN-C in (**a**) non twisted AB-AA${}^{\prime }$-AB, (**b**) non twisted AB-AA${}^{\prime }$-AB${}^{\prime }$, and (**c**) twisted T-AA${}^{\prime }$-T configuration. All bands are calculated in a $\sqrt{7}\times \sqrt{7}$ cell.

The three quadrilayer C-BN-BN-C electronic band structures are reported in Figure 2. In order to highlight the important differences near the Fermi energy, the plots are here zoomed around the Dirac point *K*, and all the calculations are performed using the same $\sqrt{7}\times \sqrt{7}$ supercell, allowing an immediate comparison.

Notably, the behavior is significantly different in the three cases.

The AB-AA${}^{\prime }$-AB stacking (left panel) shows a clear parabolic dispersion with a band gap of 51 meV and a very small (not visible) degeneracy lift off, both in valence and conduction band-edges. This is due to the (weak) interaction between the graphene layers. The AB-AA${}^{\prime }$-AB${}^{\prime }$ stacking (central panel) presents an interpenetration of the parabolic bands, with the opening of a gap at K of 0.126 eV and of two small lateral band gaps (≈7 meV) near K. The degeneracy of the bands is lifted. Finally, the bands of the twisted case (right panel) show a linear dispersion even for k-points very near to the Dirac point, with a central gap at K of 7.3 meV and two lateral gaps (at two k-points very close to K) of 0.4 meV. For the three quadrilayers, the Fermi velocity $v_{F}$ has been estimated, and the effective carrier mass $m^{*}$ calculated from the energy gap $E_{g}$ through Equation [14]:

(1) $$m^{*}=\frac{E_{g}}{2v_{F}^{2}}$$

The results are shown in Table 3.

**Figure 3:**
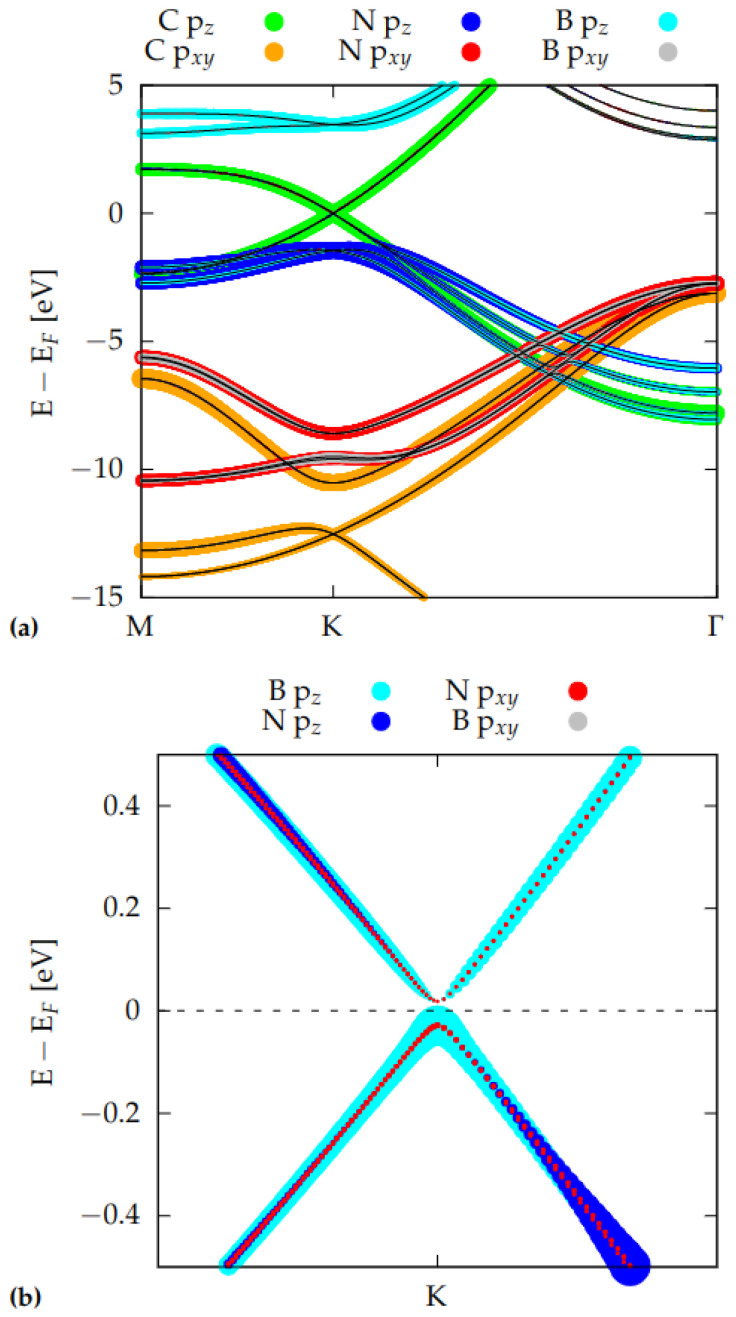
(**a**) k-resolved projected density of states (k-DOS) for the AB-AA${}^{\prime }$-AB HT. Colored dots indicate atomic orbitals; (**b**) zoom of the k-DOS near of the Dirac cone, after subtraction of the C states contribution. In both of the plots, the size of the dots is proportional to the weight of the contribution. In (**b**), the dot size is enlarged by a 200 factor in order to make them clearly visible.

In order to explain the different electronic properties of the tetralayers, it is useful to analyze the **k**-resolved Projected Density of States (k-PDOS) shown in Figure 3 calculated in a 1 × 1 unit cell along the M-K-Γ path, for the AB-AA${}^{\prime }$-AB HT.

Green and orange points in the figure are associated to $p_{z}$ and $p_{xy}$ orbitals of the carbon atoms, whereas red, blue, cyan, and grey ones are due to $p_{z}$ and $p_{xy}$ orbitals of B and N atoms.

**Figure 4:**
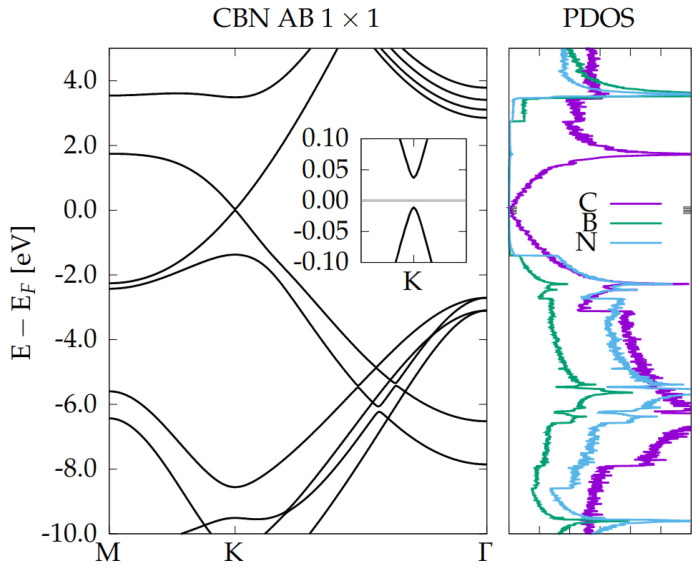
Electronic band structure and Projected Density of States (PDOS) of CBN bilayer in AB stacking in 1×1 unit cell. Inset: zoom at the Dirac point showing the opening of a small band gap.

From Figure 3a, it is clear that HT’s band structure can be roughly considered as the sum of graphene and BN-BN AA${}^{\prime }$ bilayer bandstructures. (compare with Figure S1 in the Supplementary Material) and, as expected, the Dirac cone seems to be entirely due to C $p_{z}$ orbitals. However, zooming on the region near the Dirac point (see Figure 3b), and subtracting the C orbitals contribution, not only a small band gap opening and a parabolic dispersion become evident, but also a very weak contribution of B $p_{z}$ and, to a minor extent, of N $p_{z}$ orbitals, are unveiled. In other words, the band structure of the C-BN-BN-C near K is not just the sum of those of the starting monolayers, but there is a (weak) interaction between the layers, which results in a very small hybridization of C with B and N $p_{z}$ orbitals. This interaction makes the C atoms non-equivalent and causes the opening of the small gap of 51 meV at the Dirac point (visible in Figure 2a, and in Figure 3b). [53]. In order to complete our analysis, we report in Figure 4 the band structure and the corresponding PDOS of a C-BN bilayer with AB stacking. As for the AB-AA${}^{\prime }$-AB HT case, a parabolic band dispersion is found with a very similar band gap opening of ∼49 meV.

By comparison with Figure 3, we conclude that, in the AB-AA${}^{\prime }$-AB HT, the presence of one (C-BN) or two (C-BN-BN-C) BN layers mainly influences the electronic properties far from the Fermi energy, while the low energy band dispersion and gap opening can be already explained by the interaction of one graphene with one single BN layer.

**Figure 5:**
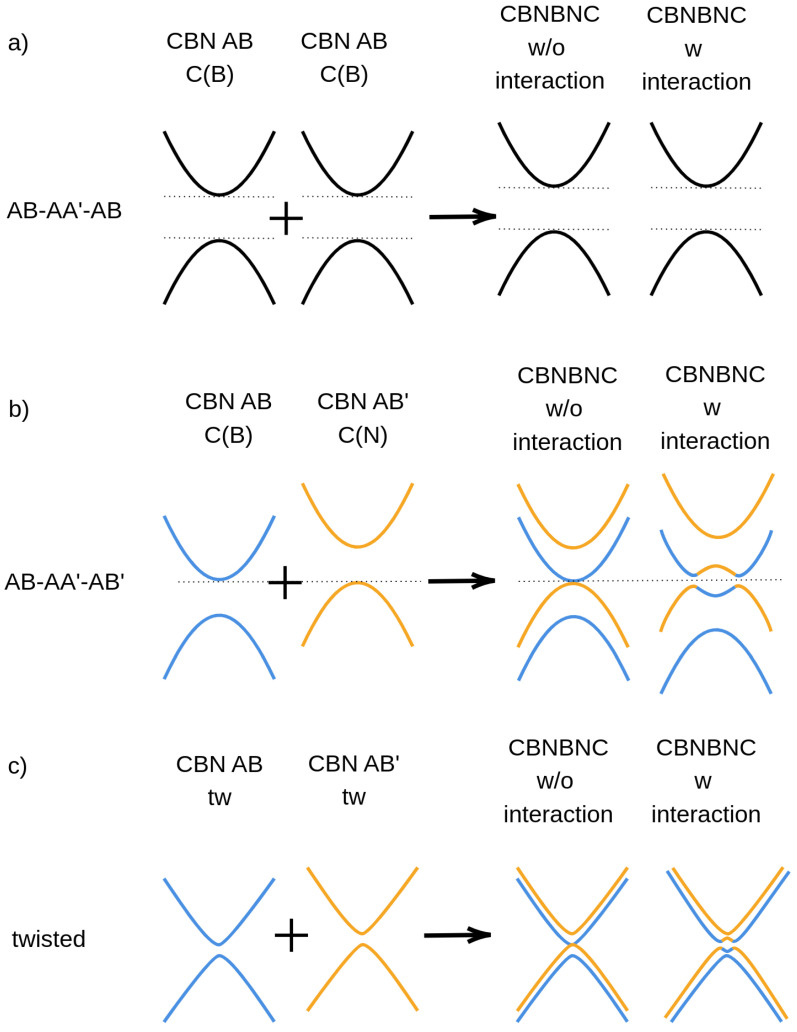
Schematic representation of the contribution of the C-BN bilayers energy states to the band structure of C-BN-BN-C quadrilayers. In the figure, we show both cases with and without the interlayer interaction for the different configurations: AB-AA${}^{\prime }$-AB (**a**), AB-AA${}^{\prime }$-AB${}^{\prime }$ (**b**) and twisted (**c**). C(B) script highlights the fact that in the AB stacking a B atom is on top of a C atoms, whereas C(N) indicates that in AB${}^{\prime }$ stacking a N atom is on top of a C atom.

A simple explanation of the low energy bands dispersion of all the quadrilayers, reported in Figure 2, can be extracted using first-neighbor 2 × 2 and 4 × 4 tight-binding (TB) models (see Supporting Information for a complete discussion). The 2 × 2 TB hamiltonian describes the C-BN bilayer, modeling it as a single layer of graphene with two non-equivalent carbon sites; the 4 × 4 TB matrix describes the quadrilayer as two layers of interacting graphene, both of them made up of two non-equivalent carbon sites. The quadrilayer TB solutions are schematically depicted in Figure 5. In the AB-AA${}^{\prime }$-AB HT case (Figure 5a), the parabolic band dispersion and gap opening are practically identical to those found in each isolated bilayer (in this case, both bilayers having AB stacking). The interaction between the layers has only a minor role and does not change the qualitative behavior. In the AB-AA${}^{\prime }$-AB${}^{\prime }$ HT, where two non-equivalent bilayers (AB and AB${}^{\prime }$) are present, the corresponding parabolic bands are shifted down (AB) and up (AB${}^{\prime }$). When the bilayers get closer, the parabola interpenetrate due to the build up of higher total dipole moment at the interface. Moreover, due to the small but not negligible interaction between the bilayers, two hybridization gaps open at the left and at the right of K, at the degenerate crossing points (see Figure 5b). Finally, in the twisted configuration, the situation is intermediate: while in AB (AB${}^{\prime }$) C-BN bilayer half of C atoms has a B(N) as vertical first neighbor, the 21.8${}^{\circ }$ rotation angle introduces several different vertical stackings, globally reducing the interaction with BN (see Figure 5c) and recovering a linear band dispersion behavior very close to the Dirac points.

In order to capture from ab-initio calculations, how the electronic bandstructure of AB-AA${}^{\prime }$-AB${}^{\prime }$ HT quadrilayer evolves from very distant AB and AB${}^{\prime }$ separated bilayers to the real composite, we perform several calculations decreasing the distance (from very large to the real equilibrium distance) between the two bilayers C-BN, using a supercell with very large size along *z*. Figure S5 of Supplementary Materials shows how, by decreasing the distance between the bilayers, the two parabolas start to compenetrate, because of the intrinsic dipoles, present in both the separated bilayers, sum up. In other words, the net effect of decreasing the distance between the AB and AB${}^{\prime }$ bilayers is to increase the intrinsic electric field (which causes a vertical shift of the parabolas) and to switch on the inter-bilayer interaction (which causes the opening of the small lateral gaps).

#### Doping and Electric Field

To complete our study, we discuss, for the AB-AA${}^{\prime }$-AB and T-AA${}^{\prime }$-T HTs, how vertical gate voltage and doping of the external graphene layers can modulate their electronic properties.

**Figure 6:**
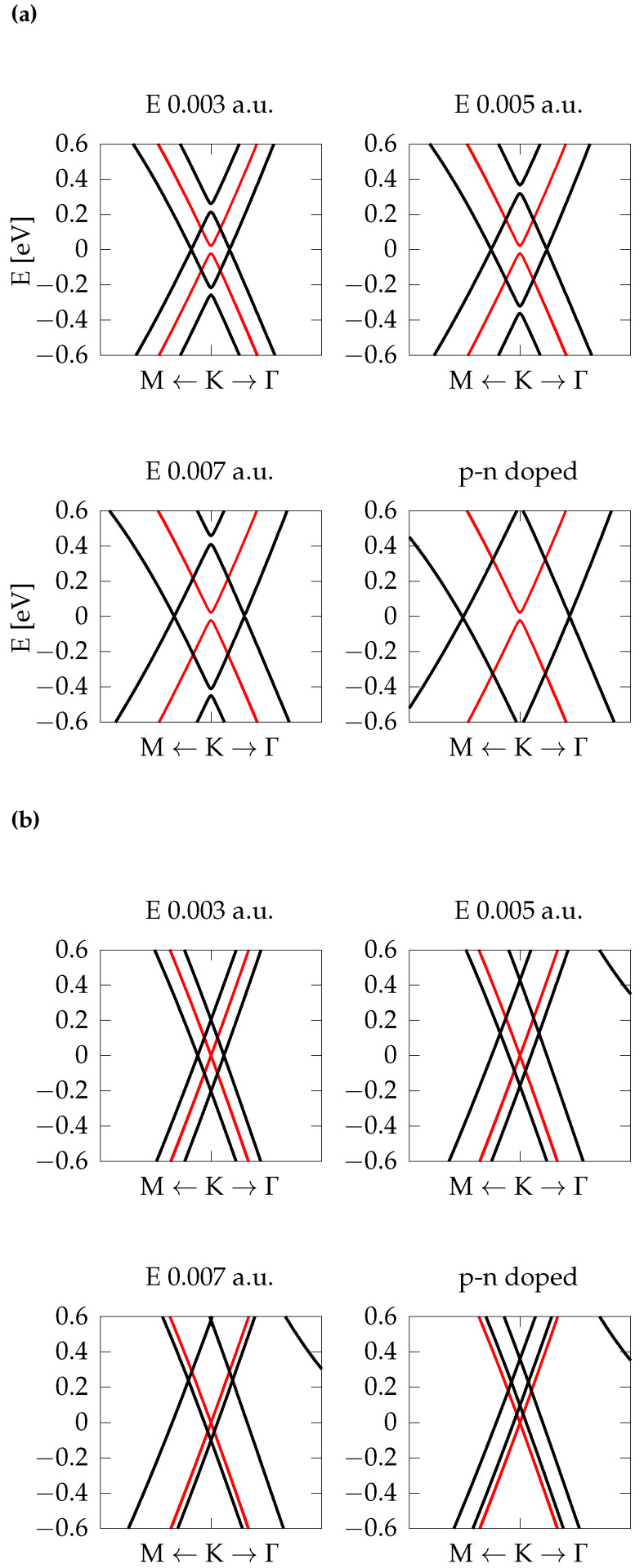
Black lines: The effect of three different external electric fields in a.u. and p-n doping on the band structure of C-BN-BN-C in (**a**) AB-AA${}^{\prime }$-AB configuration and (**b**) twisted configuration. With red lines, we report results without electric field as a reference. The zero of the energy is set at the Fermi level. The scale on the *x*-axis in (**a**,**b**) is not the same, hence the dissimilar slope of the bands does not indicate variations in the Fermi velocity of the carriers (see Table 3).

Three different electric fields, with strengths of 0.003, 0.005, and 0.007 a.u. (corresponding to 0.15,0.26, and 0.36V/Å) perpendicular to the xy plane of graphene and BN layers, have been chosen and applied to AB-AA${}^{\prime }$-AB and T-AA${}^{\prime }$-T HTs, while the p-n doping has been implemented using ad-hoc modified carbon pseudopotentials, as discussed in Section 2. The band structures near *K* (black lines), compared with the corresponding ones obtained without an electric field (red lines), are shown in Figure 6. In the presence of an external field, the Dirac cones split (the degeneracy is lifted) and interpenetrate, displacing new small band gaps on either side close to the K point of 1BZ. The amount of the cones interpenetration is proportional to the magnitude of the electric field (see Table 4).

HTs as the ones studied in the present manuscript are usually deposited on a substrate; therefore, it often happens that one of the two interfaces is doped with respect to the other one due to impurities present in the environment. For this reason, we investigate the effect of doping on one of the two carbon layers of the AB-AA${}^{\prime }$-AB HT. We found that doping induces a strong interpenetration of the Dirac cones. In fact, in this system, the doping is quite large (all C atoms in the top layer have been substituted with C${}_{n}$ pseudoatoms, and all C atoms in the bottom layer have been substituted with C${}_{p}$ atoms.) By exploiting the linear trend between the gap at K and the applied field, we can estimate that p-n-doping has, on the AB-AA${}^{\prime }$-AB HT, a net effect similar to an applied electric field of magnitude 0.01 a.u.

The case of the twisted HT is more complicated. Although the gap at K increases with the strength of the field, no clear linear trend is observed. Anyway, a rough linear fit gives an electric field of about 0.002 a.u. This field is smaller than the value obtained for the co-doped AB-AA${}^{\prime }$-AB HT, in agreement with the fact that, in the twisted quadrilayer, less than one third of the C atoms have been substituted with the pseudo-atoms C${}_{p}$ and C${}_{n}$. A further rough estimation, performed considering the four-layers system as a planar capacitor, confirms this order of magnitude for the electric field.

Finally, we notice that in the twisted case the Fermi level shifts downwards under the effect of the applied external electric field, and upon doping. This shift is due to the presence of an empty band (visible in the upper right corners in Figure 6b), which is very sensitive to perturbations, and becomes occupied for increasing electric fields. For what concerns the small lateral gaps on the left and on the right of the K point, we report in Table 4 their values. Their amplitudes do not show a linear trend with the applied electric field. They are in the range of few meV and below, hence it is interesting for possible generation and detection of THz radiation.

## 4. Conclusions

We have performed a comprehensive study of the structural and electronic properties of C-BN-BN-C quadrilayers, for three different configurations obtained by different stacking and by twisting. The most stable structure results in being the AB-AA${}^{\prime }$-AB, but all possess a negative stacking energy, suggesting that all of them can be experimentally obtained. Important differences arise in the electronic band structures near the Fermi energy, which can be traced back to the different first neighbor interaction of the non-equivalent carbon atoms in each graphene plane. Moreover, we have investigated the effect of an external electric field on these HTs. Our results show that it is possible to finely tune the band gap of a graphene/BN multilayered heterostructure by applying an external electric field perpendicular to the atomic planes. Finally, we investigate the presence of an asymmetric p-n doping on single carbon layer, which is expected in experimental realization of these HTs due to the presence of impurities, induced by disorder or edges [54]. We found that asymmetric doping acts as an effective electric field on these HTs, and this means that its effect can be compensated by an opposite electric field to restore the original band structure of the HT.

Our work suggests a possible use of graphene/BN multilayers as a detector of THz radiation, by playing with stacking order, number of BN layers, twist angle, external gate voltage, and selective doping.

## Figures and Tables

**Table 1:** Stacking energy $\Delta E^{st}$ calculated for quadrilayers and bilayers, obtained with PBE + vdW exchange correlation functional.

System	Stacking	$\Delta E^{\mathrm{st}}\left[\mathrm{meV}/\mathrm{atom}\right]$
	AB-AA${}^{\prime }$-AB	−57.8
C-BN-BN-C	AB-AA${}^{\prime }$-AB${}^{\prime }$	−34.7
	T-AA${}^{\prime }$-T	−34.3
C-BN	AB	−42.6
C-BN	AB${}^{\prime }$	−18.7
BN-BN	AA${}^{\prime }$	−22.7

**Table 2:** Lattice parameters and distances between the layers in the systems studied. The lattice constant of the twisted $\sqrt{7}\times \sqrt{7}$ HT (a = 6.55 Å) corresponds to an effective 1×1 lattice constant of about 2.47 Å. Calculations are done using PBE-vdW b86r functional.

	System	Stacking	a [Å]	d_C-BN(bottom)_ [Å]	d_BN-BN_ [Å]	d_C-BN-C(top)_ [Å]
4L	C-BN-BN-C	AB-AA${}^{\prime }$-AB	2.49	3.23	3.24	3.23
		AB-AA${}^{\prime }$-AB${}^{\prime }$	2.48	3.29	3.26	3.42
		T-AA${}^{\prime }$-T	6.55	3.40	3.23	3.40
2L	C-BN	AB	2.49	3.28		
	C-BN	AB${}^{\prime }$	2.48	3.44		
	BN-BN	AA${}^{\prime }$	2.52		3.30	
1L	Graphene	AB	2.46			
	hBN	AA${}^{\prime }$	2.50			

**Table 3:** Fermi velocities and electron-hole effective masses for the C-BN-BN-C quadrilayers.

System	$v_{F}$		m${}^{*}$
	$[10^{5}$ m/s]		[10${}^{-3}$ m${}_{e}$]
	M → K	K → Γ	
AB-AA${}^{\prime }$-AB	7.71	8.01	7.3
AB-AA${}^{\prime }$-AB${}^{\prime }$	7.82	8.40	0.9
T-AA${}^{\prime }$-T	8.20	8.14	0.05
Graphene	8.30	8.40	0

**Table 4:** Electronic gaps at K for the quadrilayers. In addition, the values of the small lateral gaps E${}_{\mathrm{gap}}^{\mathrm{lat}}$ are reported. Electric field strengths are in a.u.

C-BN-BN-C	E${}_{\mathrm{gap}}^{\left(K\right)}$	E${}_{\mathrm{gap}}^{\mathrm{lat}}$
**Stacking**	**[eV]**	**[meV]**
AB-AA${}^{\prime }$-AB		
Pristine	0.051	–
$\vec{E}$ 0.003	0.430	8.0
$\vec{E}$ 0.005	0.639	3.8
$\vec{E}$ 0.007	0.819	5.8
doped	1.245	7.3
T-AA${}^{\prime }$-T		
pristine	0.0073	0.4
$\vec{E}$ 0.003	0.406	0.1
$\vec{E}$ 0.005	0.599	2.0
$\vec{E}$ 0.007	0.681	1.8
doped	0.261	3.1
AB-AA${}^{\prime }$-AB${}^{\prime }$	0.126	6.7

## Data Availability

Not applicable.

## Supplementary Materials

The following supporting information can be downloaded at: https://www.mdpi.com/article/10.3390/nano12122118/s1, Figure S1: Band structure of graphene monolayer and 2D-BN bilayer AA${}^{\prime }$ in 1×1 unit cell.; Figure S2: Band structure of C-BN-BN-C AB-AA${}^{\prime }$-AB in a $\sqrt{7}\times \sqrt{7}$ supercell; Figure S3: Plot of the homo-1, homo, lumo, lumo+1 level at K for CBNBNC in a) AB-AA${}^{\prime }$-AB and b) twisted case; Figure S4: Electronic band structure and projected density of states of CBN bilayer in AB stacking (lower panel) and in twisted configuration (upper panel); Figure S5: Band structure calculations performed for C-BN-BN-C in AB-AA${}^{\prime }$-AB${}^{\prime }$ configuration varying the distance between the two C-BN AB and AB${}^{\prime }$ bilayers; Figure S6: Plot of the two energy eigenvalues $E_{\pm }$ of the 2×2 matrix for the C-BN bilayers; Figure S7: Plot of the four energy eigenvalue of the 4×4 matrix for AB-AA${}^{\prime }$-AB stacking evaluated for different values of Δϵ(a), *V*(b) and $t_{0}$(c) with $\Delta \epsilon^{\left(2\right)}>\Delta \epsilon^{\left(1\right)}>0$, $V^{\left(2\right)}>V^{\left(1\right)}>0$ and $t_{0}^{\left(2\right)}>t_{0}^{\left(1\right)}>0$.
