# Supplementary material for: Ab Initio Study of Graphene/hBN Van der Waals Heterostructures: Effect of Electric Field, Twist Angles and p-n Doping on the Electronic Properties
Source: Nanomaterials (Basel). 2022 Jun 20;12(12):2118. doi: 10.3390/nano12122118 (PMC9228424; doi:10.3390/nano12122118)
Supplement: Supplementary file 1 [file nanomaterials-12-02118-s001.zip › nanomaterials-1752195-supplementary.pdf]

Supplementary Material for *Ab initio* study of graphene/hBN Van der Waals heterostructures: effect of electric field, twist angles and p-n doping on the electronic properties"

Simone Brozzesi,[1,*] Claudio Attaccalite,[2] Francesco Buonocore,[3] Giacomo Giorgi,[4,5] Maurizia Palummo,[1] and Olivia Pulci[1,†]

[1] *Dipartimento di Fisica, and INFN, Universitá di Roma Tor Vergata, Via della Ricerca Scientifica 1, 00133 Rome, Italy*
[2] *CNRS/Aix-Marseille Université, Centre Interdisciplinaire de Nanoscience de Marseille UMR 7325 Campus de Luminy, 13288 Marseille cedex 9, France*
[3] *ENEA Casaccia*
[4] *Department of Civil & Environmental Engineering (DICA), Universitá degli Studi di Perugia, Via G. Duranti 93, 06125 Perugia, Italy*
[5] *CNR-SCITEC, 06123 Perugia, Italy.*

(Dated: June 20, 2022)

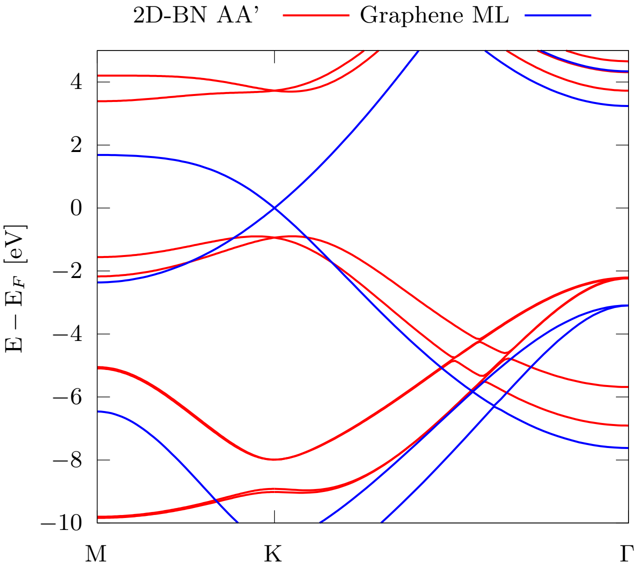

FIG. S1. Band structure of graphene monolayer (blue line) and 2D-BN bilayer AA' (red line) in 1×1 unit cell.

I. ELECTRONIC PROPERTIES IN DFT

In Fig. S1 the band structures of graphene monolayer and 2D-BN AA' bilayer are shown. Graphene is clearly a gapless semimetal, with the typical Dirac cone at the K point of the 1BZ; on the other hand 2D-BN AA' is a wide band gap insulator, as well as the starting BN monolayer. The comparison between the band structures of the separated systems with that of the C-BN-BN-C 1×1, as in Fig. 3a of the manuscript shows that the bands dispersion of the heterostructure is very similar to the direct superimposition of the bands structures of the starting systems.

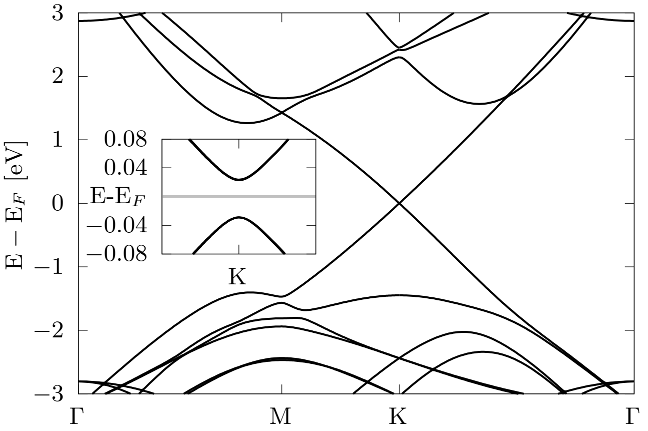

FIG. S2. Band structure of C-BN-BN-C AB-AA'-AB in a $\sqrt{7} \times \sqrt{7}$ supercell. In the inset the opening of a small band gap around 51 meV at the K point of the 1BZ of graphene is shown

Fig. S4 shows that the bands structures of the C-BN bilayers are almost identical in twisted and non twisted case, with minor differences in the valence states between −3.0 eV and −1.0 eV. In this region the projected density of states confirms a mixed contribution by the p states of C, B and N, whereas C character is strongly predominant near the Fermi level. In the AB non twisted case there is a single strong peak in the PDOS, while in that of the twisted structures a more complex set of peaks can be observed. This difference can be explained looking at the geometry of CBNBNC twisted in Fig. 1 of the manuscript: the rotation angle between the graphene and the BN layers introduces several degrees of superimposition and interaction between C and B, breaking the equivalence of all the C/B overlap present in the AB, where half of the C atom are on top of B atom, and the other half is on top of the hollow center of the hexagon of the BN layer. The effect of the different interaction scheme can be observed zooming near the K point of the

* simone.brozzesi@roma2.infn.it
† olivia.pulci@roma2.infn.it

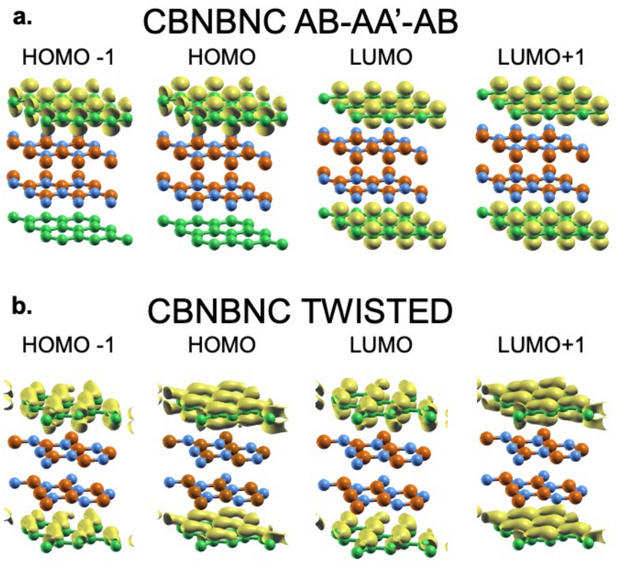

FIG. S3. Plot of the homo-1, homo, lumo, lumo+1 level at K for CBNBNC in a) AB-AA'-AB and b) twisted case.

1BZ.

II. TIGHT BINDING

The low energy band dispersion of C-BN bilayers with AB and AB' stacking, can be obtained solving a simple 2×2 tight-binding model (Eq. 1), which describes a single layer of graphene, but with two non-equivalent carbon sites (C and C*) that is:

$$\begin{pmatrix} \Delta\epsilon & tf(\mathbf{k}) \\ tf^*(\mathbf{k}) & 0 \end{pmatrix} \quad (1)$$

In Eq. 1 $\Delta\epsilon$ is an energy term taking into account the non-equivalence of the two C atoms of the basis induced by the BN layer, and $tf(\mathbf{k})$ is the first-neighbors interaction term. From the geometry of the graphene lattice, being a the C-C distance, it follows that:

$$tf(\mathbf{k}) = t\left(\exp\left(ik_x\frac{a}{2}\right)2\cos\left(\frac{k_y}{2}\sqrt{3}a\right) + \exp\left(-ik_x a\right)\right) \quad (2)$$

from which it follows that $f(\mathbf{K}) = f(\mathbf{K}') = 0$; for $k = K + q$ ($k = K' + q$), by using a Taylor expansion, it can be shown that $f(k) = \frac{3a}{2}(q_x + iq_y)(f(k) = \frac{3a}{2}(q_x - iq_y)))$.

Diagonalizing the 2×2 matrix two energy eigenvalues are found:

$$E_{\pm}(\mathbf{k}) = \frac{1}{2}\left(\Delta\epsilon \pm \sqrt{\Delta\epsilon^2 + 4t^2|f(\mathbf{k})|^2}\right) \quad (3)$$

From the previous consideration about $f(\mathbf{K})$ and $f(\mathbf{K}')$ it follows that if the two C basis atom are equivalent

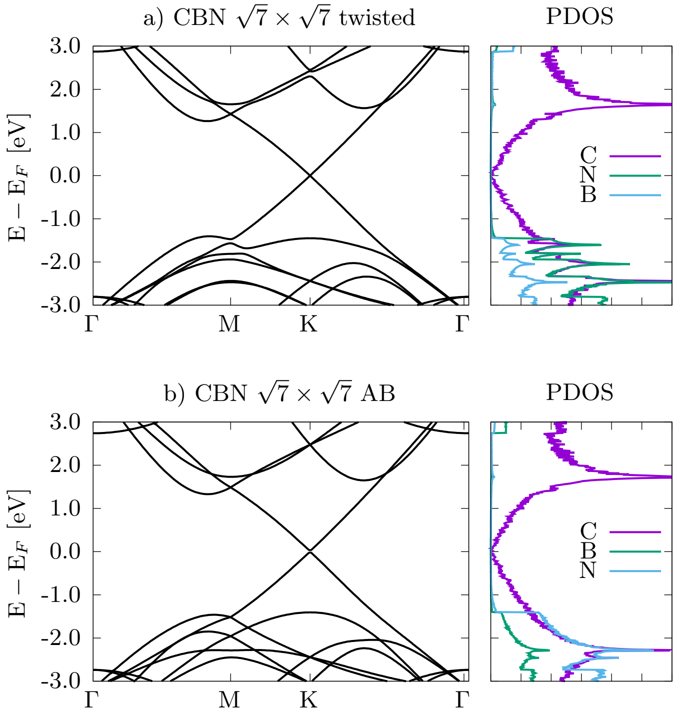

FIG. S4. Electronic band structure and projected density of states of CBN bilayer in AB stacking (lower panel) and in twisted configuration (upper panel). In both cases, a $\sqrt{7}\times\sqrt{7}$ cell was used.

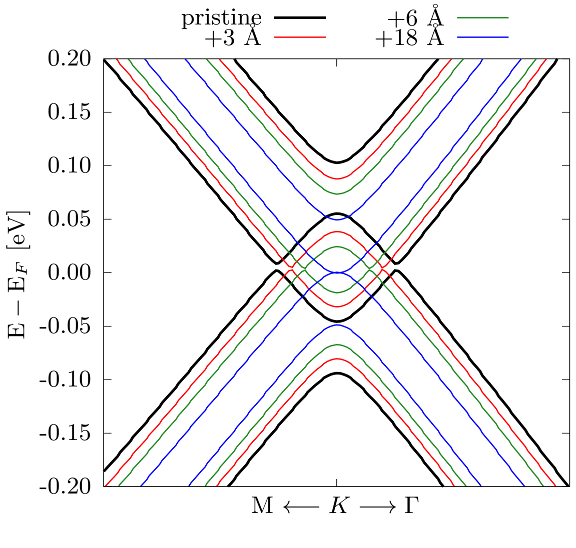

FIG. S5. Band structure calculations performed for C-BN-BN-C in AB-AA'-AB' configuration varying the distance between the two C-BN AB and AB' bilayers. Four cases have been considered: pristine, (that is, with the two C-BN at the equilibrium distance), and with the C-BN distance increased by 3 Å, 6 Å and 18 Å respectively.

($\Delta\epsilon = 0$), as in single layer graphene, the energy gap closes at the K,K′ points of the 1BZ with the well

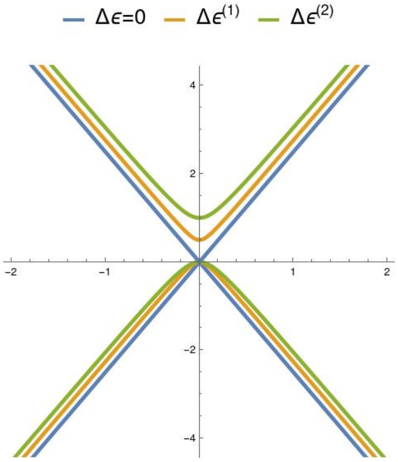

FIG. S6. Plot of the two energy eigenvalues $E_\pm$ of the 2×2 matrix for the C-BN bilayers. The eigenvalues have been calculated considering $\Delta\epsilon^{(2)} > \Delta\epsilon^{(1)} > 0$, in order to simulate an increasing degree of non-equivalence of the two carbon atoms in the basis, i.e. a stronger interaction with the BN sheet in the bilayer.

known Dirac cones dispersion. Conversely, if the non-equivalence of the two C atoms ($\Delta\epsilon \neq 0$), as in the case of CBN bilayer, is introduced, the dispersion of the energy bands around K become parabolic, the Dirac cone disappears and a small band gap opening proportional to $2\Delta\epsilon$ takes place. This effect is shown in Fig.S6, where the two eigenvalues $E_\pm(\mathbf{k})$ are plotted for three different values of $\Delta\epsilon$, specifically $\Delta\epsilon^{(2)} > \Delta\epsilon^{(1)} > 0$. From our ab-initio calculations and previous formulas we estimate $\Delta\epsilon$=49 meV in bilayer CBN.

Different stacking order, AB (AB'), induces a negative (positive) shift of the two parabolas, associated to a positive (negative) potential due to the interaction of carbon C* atoms with first neighbours B (N).

Similarly, 4×4 models Eqs.(4,5), allow to explain the quadrilayers low energy band dispersions, considering two interacting graphene layers each of them made up of non-equivalent carbon sites.

$$\begin{pmatrix} \Delta\epsilon + V & 0 & tf(\mathbf{k}) & 0 \\ 0 & -V & t_0 & tf(\mathbf{k}) \\ tf^*(\mathbf{k}) & t_0 & V & 0 \\ 0 & tf^*(\mathbf{k}) & 0 & \Delta\epsilon - V \end{pmatrix} \tag{4}$$

$$\begin{pmatrix} \Delta\epsilon + V & tf(\mathbf{k}) & 0 & t_0 \\ tf(\mathbf{k}) & V & t_0 & 0 \\ 0 & t_0 & -V & tf(\mathbf{k}) \\ t_0 & 0 & tf(\mathbf{k}) & \Delta\epsilon - V \end{pmatrix} \tag{5}$$

Since we have different stacking sequence for the three studied systems, an electric potential term V has been introduced in the diagonal terms in the 4×4 matrices. In this way it is possible to include in the tight-binding model the contribution of a electric potential that can account for both an external electric field, as done in the AB-AA'-AB HT, and the possible presence of an internal dipole moment in the systems (as it happens in the AB-AA'-AB' HT). An off-diagonal term t_0 has also been included to take into account the interaction, mediated by the BN sheets, between the two graphene layers. In Fig. S7 the roles that these parameters play in modifying the band dispersion are shown. As expected, the effect of the non-equivalence of the C atoms represented by $\Delta\epsilon$ is the same shown in the 2×2 model (Fig. S7a). The potential term V, instead, is responsible for a splitting and a shift of the doubly degenerate valence and conduction band: it shifts down the conduction band and lifts up the valence band, resulting in the interpenetration of these states (Fig. S7b). The interlayer interaction term t_0 is responsible for the opening of the two small lateral band gap, with parabolic dispersion, in the cross regions where the band interpenetration of the bands induced by V takes place.

For AB-AA'-AB HT (left panel of Fig. 2 of the manuscript), the four parabolic solutions remain doubly degenerate while, when the interaction t_0 is turned on, a small increase of the gap with respect to the bilayer AB and a very small splitting of the bands (not visible) take place. This is schematically shown in Fig.5a of the manuscript. The band dispersion around K of AB-AA'-AB' and twisted shown in central and right panel of Fig. 2 of the manuscript are qualitatively well described by the tight-binding model proposed, with clear evidences of the impact of the internal dipole momenta and the interlayer interaction of the external graphene layers represented by the V and t_0 parameter.

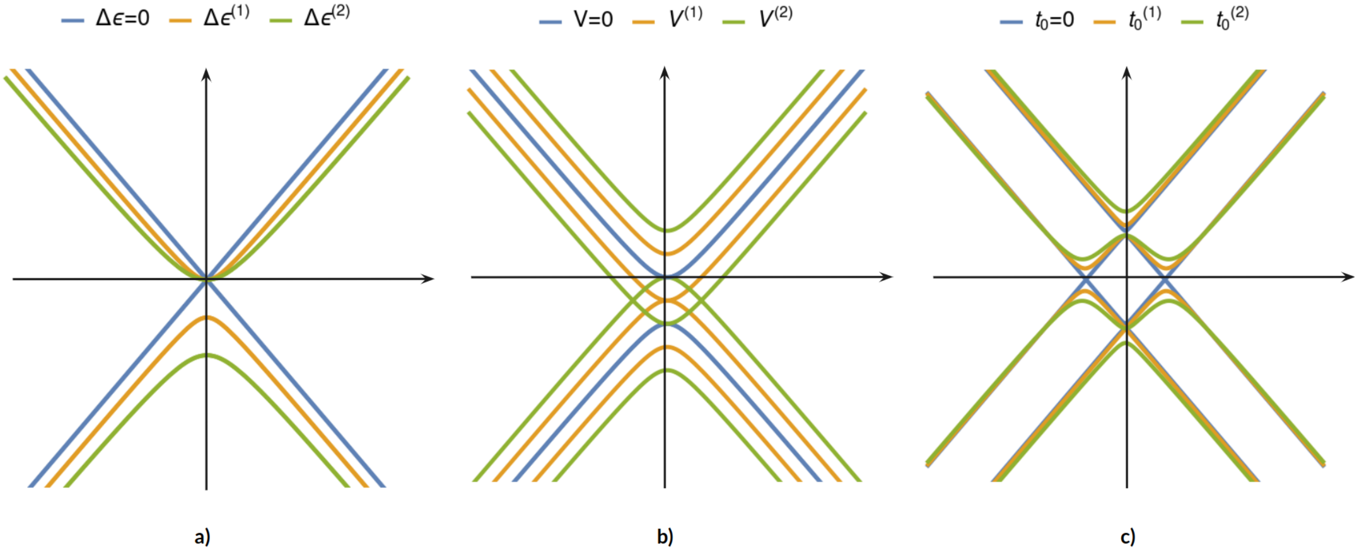

FIG. S7. Plot of the four energy eigenvalue of the 4×4 matrix (eq.4,5) for AB-AA'-AB stacking evaluated for different values of $\Delta\epsilon$(a), V(b) and t_0(c) with $\Delta\epsilon^{(2)} > \Delta\epsilon^{(1)} > 0$, $V^{(2)} > V^{(1)} > 0$ and $t_0^{(2)} > t_0^{(1)} > 0$. The aim is to observe how higher degree of non-equivalence term $\Delta\epsilon$, electric potential V and interplanar interaction t_0 affect singularly the dispersion of the energy eigenvalues
